# One-minute preceptor as a microskills teaching and learning model in operative dentistry clinical settings: a student perspective

**DOI:** 10.7717/peerj.21461

**Published:** 2026-07-08

**Authors:** Azhar Iqbal, Kiran Kumar Ganji, Saeed Alqahtani, Muhammad Sohail Zafar, Osama Khattak, Yasir Dilshad Siddiqui, Muhammad Rizwan Memon, Afaf A. Almabadi, Mohmed Isaqali Karobari

**Affiliations:** 1 Department of Restorative Dental Sciences, College of Dentistry, Al Jouf University, Sakaka, Saudi Arabia; 2 Department of Preventive Dentistry, College of Dentistry, Al Jouf University, Sakaka, Saudi Arabia; 3 Institute of Health Professions Education, Sri Balaji Vidyapeeth, Pondicherry, Tamil Nadu, India; 4 Department of Research Analytics, Saveetha Dental College & Hospitals, Saveetha Institute of Medical and Technical Sciences, Saveetha University, Chennai, Tamil Nadu, India; 5 Department of Clinical Sciences, College of Dentistry, Ajman University of Science & Technology, Ajman, United Arab Emirates; 6 Center of Medical and Bio-allied Health Sciences Research, Ajman University of Science & Technology, Ajman, United Arab Emirates; 7 School of Dentistry, University of Jordan, Ajman, Jordan; 8 Department of Prosthodontics, College of Dentistry, Aljouf University, Sakaka, Saudi Arabia; 9 Department of Oral & Maxillofacial Prosthodontics, Faculty of Dentistry, King Abdulaziz University, Jeddah, Saudi Arabia; 10 Department of Conservative Dentistry and Endodontics, Saveetha Dental College and Hospital, Saveetha Institute of Medical and Technical Sciences, Saveetha University, Chennai, Tamil Nadu, India

**Keywords:** One minute preceptorship, Microskills, Teaching, Learning

## Abstract

**Background:**

Clinical teaching of dentistry often takes place in busy patient-care environments, where there may not be sufficient time for real-time supervision or feedback, compromising student learning during clinical encounters. The One-Minute Preceptor (OMP) is a structure microskills model of teaching which has been developed for efficient clinical teaching in brief encounters. Accordingly, this study aimed to evaluate the OMP model as a microskills teaching and learning approach in operative dentistry clinical settings, with particular emphasis on student learning, reasoning, clinical performance, and perceptions of its usefulness in busy clinical environments.

**Methods:**

A randomized controlled educational intervention with a two-group pretest-posttest design was conducted among third-year Bachelor of Dental and Oral Surgery students. Fifty students who provided written informed consent were included in the study. Using simple randomization with a random number table, participants were allocated to either a control group (*n* = 26) receiving traditional clinical teaching or an intervention group (*n* = 24) receiving teaching based on the One-Minute Preceptor model. Student perceptions of the OMP model were collected using a structured 5-point Likert-scale questionnaire.

**Results:**

Following the intervention, students in the OMP group exhibited significant improvement across nearly all assessed domains, whereas the control group showed either minimal gains or slight declines in several areas. Post-intervention ratings showed statistically significant improvements (*p* < 0.01) across most items, particularly within the domains of Commitment, Probe, Feedback, and Overall Competence. Students in the intervention group reported high satisfaction with the OMP model, with most rating it as beneficial for learning in restorative dentistry clinical sessions.

**Conclusion:**

The findings of this study suggest that the OMP model may be a useful teaching and learning approach for operative dentistry clinical settings, with improvement observed in several assessed student performance domains and positive student perceptions regarding its applicability in busy clinical environments. Further studies with larger samples and more robust statistical analyses are warranted.

## Introduction

Clinical teaching of dentistry often takes place in busy patient-care environments, where there may not be sufficient time for real-time supervision or feedback, compromising student learning during clinical encounters. In operative dentistry, the challenges are essential since undergraduate students must combine clinical reasoning, communication and procedural skills, all while consulting real patients and obtaining the clinical requirements (Ferenchick et al., 1997; Irby, 1995; Schéle, Hedman & Hammarström, 2011). To maximize the educational value of short chairside interactions without interruption of patient care, structured teaching methods are therefore needed (Bowen & Irby, 2002; Weitzel, Walters & Taylor, 2012).

The One-Minute Preceptor (OMP) is a structure microskills model of teaching described by Neher et al. (1992) which has been developed for efficient clinical teaching in brief encounters. This process is composed of five fundamental stages: getting a commitment—the learner is encouraged to express their opinion, diagnosis or management decision. The preceptor asks the learner to justify their answer so he can assess the depth of their knowledge. Highlight important rules that are related to the case being tried. The preceptor identifies and reinforces effective behaviors and strategies learners use during the clinical placement (Neher et al., 1992; Neher & Stevens, 2003). OMP has been widely discussed as a practical and efficient teaching model for busy clinical settings (Bowen & Irby, 2002; Irby, 1992; Irby, 1995).

Research on OMP in medicine and other health professions has reported benefits in focused teaching, feedback, and clinical reasoning, and favorable learner perceptions have been described in pediatrics, radiology, nursing, pharmacology, and obstetrics and gynecology (Chandra et al., 2018; Furney et al., 2001; Gatewood & De Gagne, 2019; Griffiths et al., 2022; Irby, Aagaard & Teherani, 2004; Iyer, Nanditha & Raman, 2017; Kachewar, 2015; Shambharkar et al., 2021; Sharma et al., 2023). Recent literature suggests that OMP continues to be actively explored in contemporary clinical education, but predominantly outside dentistry. For example, recent studies have examined the applicability of OMP in nursing preceptorship and identified both facilitators and barriers to its implementation in practice settings, highlighting the continuing relevance of the model in workplace-based education (Liu et al., 2025). In undergraduate medical education, randomized comparative work has shown that OMP can improve clinical reasoning outcomes relative to traditional teaching, although alternative structured models such as SNAPPS may perform better for some reasoning-related outcomes in simulated settings. More recent intervention studies have also reported improved learner performance and satisfaction when OMP is combined with other structured teaching approaches, such as flipped-classroom methods, in general practice training (Yueyang, Li & Haiyan, 2025). These recent studies indicate that OMP remains an evolving area of educational research; however, most of this work has focused on medicine and nursing rather than discipline-specific dental procedural training. This makes the evidence base in operative dentistry comparatively limited and reinforces the need for context-specific studies in undergraduate dental education.

In dental education, Sakaguchi (2010) described the adaptation of OMP in a teaching clinic to enhance preceptor–student communication and evidence-based clinical decision-making. More recently, OMP has also shown feasibility in radiology education, where it demonstrated a trend toward greater improvement than conventional teaching, although the differences were not statistically significant (Salakay et al., 2025). However, the available literature remains limited in dental education, particularly in operative dentistry clinical training. Much of the published evidence on OMP has emerged from medicine and nursing, while relatively fewer studies have examined its application in discipline-specific dental procedures. In addition, recent stronger comparative designs have largely been reported outside dentistry. Operative dentistry requires students to develop stepwise procedural competence alongside clinical decision-making in real-time chairside settings, making it distinct from more general clinical teaching contexts. Given the increasing need for efficient, feedback-oriented teaching strategies in high-volume undergraduate clinical settings, this represents a meaningful and timely gap in the literature. Accordingly, the suitability of OMP for operative dentistry procedures, particularly from the student perspective, warrants further investigation. Given the increasing need for efficient, feedback-oriented teaching strategies in high-volume undergraduate clinical settings, this represents a meaningful and timely gap in the literature. Accordingly, the suitability of OMP for operative dentistry procedures, particularly from the student perspective, warrants further investigation.

This study aimed to evaluate the OMP model as a microskills teaching and learning approach in operative dentistry clinical settings, with particular emphasis on student learning, reasoning, clinical performance, and perceptions of its usefulness in busy clinical environments. In doing so, this study contributes discipline-specific evidence on the use of OMP in undergraduate operative dentistry education. The primary research question of this study was whether the One-Minute Preceptor model, when used in operative dentistry clinical teaching, leads to better student learning, clinical performance, and student perceptions compared with traditional clinical teaching. It was hypothesized that students exposed to the OMP model would demonstrate greater improvement in assessed operative dentistry competencies and more favorable perceptions of the teaching-learning experience than students receiving traditional teaching.

## Methodology

### Study setting & design

This study was designed as a randomized controlled educational intervention with a two-group pretest–posttest design, conducted during the operative dentistry clinical sessions of third-year Bachelor of Dental and Oral Surgery students at the College of Dentistry, Jouf University, from August 2023 to January 2024. Ethical approval was obtained from the Local Committee of Bioethics for Research (Approval No. 1-09-43).

### Participants and allocation

All eligible third-year Bachelor of Dental and Oral Surgery (BDS) students posted in the operative dentistry clinics during the study period were invited to participate. Written informed consent was obtained from the enrolled students. Fifty students were included in the study. Participants were allocated into either the control group (*n* = 26), which received traditional clinical teaching, or the intervention group (*n* = 24), which received teaching based on the One-Minute Preceptor model, using simple randomization with a random number table.

### Study procedure

The study was conducted in the following phases:

#### Phase 1: choosing competencies and creating evaluation standards

The competencies assessed in the present study are taken from the operative dentistry curriculum meant for third-year BDS students. They are in line with the learning objectives of cavity preparation and direct composite restoration procedures taught during the period of study. The competencies were chosen with respect to their relevance to chairside clinical performance, their importance in undergraduate operative dentistry training, and their capacity to be observed and assessed during short clinical sessions. Evaluation criteria were subsequently created for each competency to standardize assessment pre- and post-intervention for both study groups. To assess the selected competencies’ knowledge and clinical application the pre-test and post-test instruments were developed.

#### Phase 2: pre-test, post-test, and feedback questionnaire were developed and validated

The pre-test and post-test instruments were designed to assess knowledge and clinical application related to the selected competencies. The feedback questionnaire was developed through a structured process that included literature review, item drafting, expert review for content validity, pilot testing, refinement of ambiguous items, and assessment of reliability and validity.

#### Phase 3: pretesting and sensitization

A total of four faculty members involved in operative dentistry clinical teaching participated in the orientation and training session for implementation of the OMP model. Students and faculty members were oriented to the OMP paradigm through a one-day training session conducted at the College of Dentistry, Jouf University. During this session, a 30-minute didactic lecture was delivered by the lead investigator, followed by a 30-minute interactive discussion and role play to ensure that the OMP teaching and learning model was properly understood and that the five microskills were clearly explained. At the end of the session, participating faculty members were further guided on providing feedback

#### Phase 4: allocation of the students into two groups

After the pretest evaluation regarding knowledge of cavity preparation and dental composite restoration, students were allocated into the control group (Group A, *n* = 26) and the intervention group (Group B, *n* = 24). Simple randomization using a random number table was adopted for group allocation.

Intervention Group: Students in the intervention group were taught using the OMP model during individual clinical encounters. Each teaching interaction was structured around the five microskills of OMP: (1) getting a commitment from the student regarding the diagnosis or treatment plan, (2) probing for supporting evidence by asking the student to explain the reasoning behind the response, (3) teaching a general principle relevant to the case, (4) reinforcing what was done well, and (5) correcting mistakes through immediate and focused feedback. During these brief targeted encounters, one student interacted directly with the preceptor while the remaining students observed and learned from both the clinical discussion and the feedback provided. Each clinical session lasted 120 min.

Control Group: Students in the control group received traditional clinical teaching during the same scheduled operative dentistry sessions. In this format, the tutor supervised multiple students simultaneously, provided instructions to the group as a whole, observed ongoing clinical procedures, and offered feedback in the usual manner practiced in the department. Unlike the OMP-based approach, teaching in the control group was not structured around the five microskills or individual brief preceptor-student encounters. Each clinical session lasted 120 min.

#### Phase 5: post test and evaluation

A post-test was conducted to assess immediate learning gain following the intervention period. Both groups were subjected to a final post-intervention evaluation using multiple choice questions (MCQs) and clinical scenarios based on the required competencies. The student performance was scored by two examiners independently. The average of the two scores was taken to improve inter-examiner reliability. Scholars were told that these scores would not be factored in the final summative evaluation.

#### Phase 6: feedback

A feedback questionnaire created by the authors was given to students engaged in the study to obtain their perception of the OMP teaching model using a standard 5 point Likert scale.

Development of questionnaire: The creation and validation of a questionnaire aimed at assessing perceptions of the OMP teaching modality underwent a formal procedure, which involved a number of steps. The study aims were initially established as to study the effectiveness of the teaching delivered, quality and transparency of the lecture, engagement of the student by studying, and satisfaction of the student with the lecture. A detailed literature review was carried out to find validated questionnaires and items that are likely to be relevant. A framework of question domains was developed on this basis and a combination of item types was trailed in the draft item. Experts in dental education were then consulted to review the draft to test content validity and make sure that the questions covered the areas effectively. A pilot study was conducted among a small group of students after taking expert feedback in order to check clarity, length and structure. The pilot responses were analyzed enabling item analysis. Items were deleted or modified owing to ambiguity or redundancy. For assessing reliability, use internal consistency measures such as Cronbach’s alpha and the test–retest reliability to assess the stability over time. Through statistical methods like factor analysis, the construct validity was proven and the experts input as well as comparison with standard tool confirmed the content and criterion validity. The questionnaire was finalized and prepared for use based on all comments received. The methodical research enabled the development of a reliable and valid tool to assess the perception of OMP teaching modality.

Intervention and comparison: Students in the intervention group were taught using the OMP model during individual brief chairside clinical encounters structured around the five microskills. Students in the control group underwent traditional clinical teaching during the same scheduled clinical sessions. Each session lasted 120 min for both groups.

### Outcome assessment

Baseline knowledge was assessed using a multiple-choice-question-based quiz. After completion of the intervention period, both groups underwent post-intervention evaluation using multiple-choice questions and clinical scenarios based on the required competencies. Student perceptions of the OMP model were collected using a structured 5-point Likert-scale questionnaire.

### Data analysis

Data were analyzed using SPSS version (IBM Corp., Armonk, NY, USA) 27 and Microsoft Excel 2019 (Redmond, WA, USA). Descriptive statistics were used to summarize participant characteristics and questionnaire responses. Normality of continuous variables was assessed prior to parametric testing. Within-group pre- and post-intervention comparisons were analyzed using paired Student’s *t*-test. Between-group comparisons were made using the observed mean change scores between the control and intervention groups across the assessed domains. A *p*-value of less than 0.05 was considered statistically significant for all analyses.

## Results

A total of fifty third-year Bachelor of Dental Surgery students participated in the study, with 26 students assigned to the control group (traditional teaching) and 24 students to the intervention group (OMP-based teaching). The gender distribution was nearly balanced, comprising 24 (48%) males and 26 (52%) females, ensuring gender representation without significant bias.

Baseline Evaluation (Pre-intervention): Of the 50 participating students, 24 (48%) were male and 26 (52%) were female. Baseline and post-intervention performance data for the control and intervention groups are presented in Table 1. Only two domain items, history taking skills and outline form, showed minor variations between the groups prior to the intervention. Additionally, the intervention group demonstrated slightly lower baseline ratings in the isolation and convenience form domains, suggesting a marginally weaker starting point in these procedural skills.

**Table 1:** Pre-test, post-test, and mean change scores for assessed clinical domains in the control and intervention groups.

Domain	Item	Control group			Intervention group			Mean differences in change between groups
		Pre	Post	Change	Pre	Post	Change	
Commit	Obtain informed consent	4.14	4	−0.14	4	4.08	0.08	0.22
Commit	Involve in history taking skills	4.12	4.14	0.02	4.03	4.27	0.24	0.22
Commit	Involve in intraoral examination skills	4.18	4.03	−0.15	4.07	4.19	0.12	0.27
General rules	Taught professionalism	3.77	3.64	−0.13	3.42	3.95	0.53	0.66
General rules	Taught counselling skills	3.64	3.44	−0.2	3.33	3.93	0.6	0.8
Probe	Evaluated clinical judgement organization/efficiency	3.51	3.38	−0.13	3.59	3.67	0.08	0.21
Probe	Evaluated isolation	3.96	3.94	−0.02	3.96	3.9	−0.06	−0.04
Probe	Evaluated outline form	4.12	4.22	0.1	4	4.12	0.12	0.02
Probe	Evaluated resistance form	3.64	3.44	−0.2	3.33	3.93	0.6	0.8
Probe	Evaluated retention form	3.51	3.38	−0.13	3.59	3.67	0.08	0.21
Probe	Evaluated convenience form	3.96	3.94	−0.02	3.96	3.9	−0.06	−0.04
Feedback	Explained the selection of restorative material	4.12	4.22	0.1	4	4.12	0.12	0.02
Feedback	Gave feedback on condensation of material	3.86	3.66	−0.2	3.78	3.93	0.15	0.35
Feedback	Gave feedback on finishing & polishing	4.13	4	−0.13	4.07	4.07	0	0.13
Feedback	Gave feedback on occlusion contact	3.94	3.83	−0.11	3.98	4.09	0.11	0.22
Overall	Work on disposing and post-operative instructions	4.18	4.01	−0.17	4.03	4.23	0.2	0.37
Overall	Overall clinical competence	4.18	4.01	−0.17	4.07	4.18	0.11	0.28

Post-intervention Outcomes: Following the intervention, students in the OMP group exhibited significant improvement across nearly all assessed domains, whereas the control group showed either minimal gains or slight declines in several areas. As detailed in Table 1, the most prominent improvements in the OMP group were observed in:

• Commitment domain: items related to *informed consent*, *history taking*, and *intraoral examination skills* all showed positive mean changes (0.08–0.24), indicating better student engagement and diagnostic reasoning.
• Feedback domain: items addressing *material selection*, *condensation*, and *occlusion feedback* improved consistently, with mean differences in change between groups ranging from 0.13 to 0.35.
• General rules domain: the intervention group showed notable improvement in *professionalism* and *counselling skills* (mean differences 0.66 and 0.8, respectively), highlighting OMP’s role in fostering professional and communicative competencies.

Overall, the OMP group demonstrated a consistent upward trend, whereas the control group exhibited negative or negligible changes across most domains. The intervention group showed greater improvement than the control group in several assessed domains, including overall clinical competence, based on the observed mean changes. These findings suggest a favorable effect of the OMP-based approach within this cohort.

Self-reported Perception of OMP as a Teaching Modality: The participants’ self-reported perceptions, analyzed using a 5-point Likert scale (Table 2), further supported the objective findings. Post-intervention ratings showed statistically significant improvements (*p* < 0.01) across most items, particularly within the domains of Commitment, Probe, Feedback, and Overall Competence. Key findings include:

**Table 2:** Pre-test and post-test comparison of student perception/performance items following implementation of the OMP model.

**Domain**	**Item**	**Pre**	**Post**	*P* value
Commit	I felt adequately in obtaining the informed consent from the patient about the purpose and process of the dental procedure before it was performed.	3.3	4.26	<0.01
Commit	I am confident in my ability to take a patient’s medical history accurately and effectively.	3.1	4.2	<0.01
Commit	I am proficient in conducting thorough intraoral examinations.	3.2	4.13	<0.01
General rules	The OMP teaching methods used in my dental education effectively conveyed the importance of professionalism.	3.41	3.9	0.24
General rules	My dental curriculum adequately addressed the development of effective counselling skills.	3.8	3.97	0.1
Probe	My clinical judgments and organization/efficiency skills were evaluated adequately during my dental training.	3.23	4	<0.01
Probe	I received constructive feedback on my isolation techniques and various aspects of restorative procedures.	3.15	3.9	0.21
Probe	I received constructive feedback on outline form of restorative procedure	3.45	4.34	<0.01
Probe	I received constructive feedback on resistance form of restorative procedure	3.15	4.12	<0.01
Probe	I received constructive feedback on retention form of restorative procedure	3.1	4.2	<0.01
Probe	I received constructive feedback on Convenience form of restorative procedure	3.2	3.9	0.32
Feedback	I was well-informed about the factors influencing the selection of appropriate restorative materials.	3.7	4.17	<0.01
Feedback	Instructors provided meaningful feedback on my performance in condensation of restorative materials.	3.4	4.1	<0.01
Feedback	Instructors provided meaningful feedback on my performance in finishing & polishing of restorations.	3.5	4.2	<0.01
Feedback	Instructors provided meaningful feedback on my performance in obtaining occlusion contact.	3.2	4	<0.01
Overall	I received training and evaluation on proper disposal methods and providing post-operative instructions to patients.	3.4	4	<0.01
Overall	I am confident in my overall clinical competence upon completing my dental education.	3.21	4.3	<0.01

• Significant increases in self-perceived competence in *obtaining informed consent* (mean 3.3 → 4.26), *history taking* (3.1 → 4.2), and *intraoral examination* (3.2 → 4.13), all with *p* < 0.01.
• Marked improvement in *clinical judgment*, *outline form*, *resistance form*, and *retention form* (*p* < 0.01), confirming enhanced procedural and analytical skills.
• Substantial improvement in *feedback-related domains*, including *condensation*, *finishing and polishing*, and *occlusion contact* (*p* < 0.01), underscoring the effectiveness of OMP’s real-time feedback mechanism.

In contrast, non-significant differences were found in *professionalism* (*p* = 0.24), *counselling skills* (*p* = 0.10), *isolation techniques* (*p* = 0.21), and *convenience form* (*p* = 0.32), suggesting that these aspects may require longer exposure or reinforcement for measurable improvement.

Overall Satisfaction with the OMP Teaching Model: Students reported that the structured and focused nature of OMP sessions enhanced their understanding, confidence, and ability to apply knowledge in clinical contexts. Students in the intervention group reported high satisfaction with the OMP model, with most rating it as beneficial for learning in restorative dentistry clinical sessions.

## Discussion

Often in a busy patient-care setting dental students are expected to perform more than one task at the same time while fulfilling the clinical requirements of their curriculum (Schéle, Hedman & Hammarström, 2011). In such environments, it is particularly appealing to identify a teaching approach that enables students to learn while interacting with and treating patients in real time. This type of teaching-learning activity gives the teacher an opportunity to spend one-on-one time with students, observe them directly, monitor their involvement in patient care, and guide them in authentic clinical situations (Ferenchick et al., 1997). However, because the time available with each student and patient is limited, such teaching requires a structured and efficient approach. The OMP model has emerged as a useful modality in these settings because it helps shape educational discussion within a short period of time and allows for immediate and focused feedback (Irby, Aagaard & Teherani, 2004; Neher & Stevens, 2003; Shambharkar et al., 2021).

The findings of the present study suggest that the OMP model may be a useful teaching and learning approach in operative dentistry clinical settings. Students in the intervention group showed improvement across several assessed domains, and student perceptions regarding the usefulness of OMP were also favorable. These findings are educationally relevant because operative dentistry requires not only clinical reasoning and decision-making but also the performance of sequential procedural steps in a busy chairside environment. In this context, a structured microskills-based teaching model may help preceptors organize brief interactions more effectively and support students’ learning without interrupting patient care.

One of the important educational strengths of OMP is its emphasis on immediate feedback. Instantaneous feedback helps students identify mistakes, reflect on them, and make necessary corrections during the learning process (Shambharkar et al., 2021). Feedback is widely regarded as essential for acquiring and refining specific skills and behaviors that can ultimately improve patient care, and students consistently rank feedback among the most valued components of practice-based learning (Spooner, 2025; Weitzel, Walters & Taylor, 2012). In contrast, traditional teaching methods often depend heavily on the individual teacher’s initiative and may lack a structured, methodical, and immediate feedback process. Similar gaps in traditional teaching approaches have also been noted in earlier studies (Chandra et al., 2018; Opitz, Ferdinand & Mecklinger, 2011). The present findings, especially those related to commitment and feedback-oriented domains, are therefore consistent with the broader literature suggesting that OMP enhances the feedback process during clinical teaching encounters.

The present findings are also broadly in agreement with previous studies on OMP in medicine and other health professions. Furney et al. (2001) reported that OMP, used as a brief educational intervention, improved most microskills, although “teaching general rules” showed less improvement in their study. Irby, Aagaard & Teherani (2004) observed that OMP shifted teaching toward more focused and disease-specific instruction, while Aagaard, Teherani & Irby (2004) found that preceptors using OMP were equal or superior to those using traditional approaches in identifying patients’ clinical problems and evaluating learners. Sharma et al. (2023) also demonstrated high participant satisfaction with OMP compared with the traditional model and recommended its incorporation into routine clinical teaching. Cheema, Arora & Kumar (2021) also reported that OMP was perceived as a useful pedagogical tool, particularly in improving presentation skills, feedback, assessment strategies, and motivation for further reading. These findings support the view that OMP is not merely a brief teaching technique, but a structured model that may improve the quality and efficiency of short clinical teaching encounters.

Similar findings have also been reported in other disciplines. Kachewar (2015) used OMP as a systematic approach for teaching radiology residents in ultrasonographic evaluation of acute abdominal pain and found it to be effective in helping learners reach appropriate diagnostic conclusions. Gulati (2016) reported that OMP was instrumental in improving examination skills, communication of findings, logical differential reasoning, and independent study among pathology residents. Iyer, Nanditha & Raman (2017). observed that nearly all participants considered OMP more effective than traditional teaching methods. Likewise, Harkare et al. (2013) found that OMP was a highly successful teaching-learning approach in clinical teaching for undergraduate MBBS students, with students reporting that it highlighted their strengths and weaknesses, improved the teacher-student bond, reduced fear in the learning environment, and made immediate feedback beneficial. Together, these studies reinforce the educational value of OMP across a range of health-professions settings.

The present study extends this line of work into operative dentistry, where evidence remains limited. While OMP has previously been described in dental teaching settings, very few studies have focused specifically on restorative or operative dental procedures in undergraduate clinical training (Sakaguchi, 2010). In the present study, the emphasis was on clinical procedures related to restorative dentistry in real clinical settings. This is an important distinction, because some findings in operative dentistry are procedure-specific and may not be fully comparable with studies conducted in medicine, nursing, radiology, pathology, or other non-dental fields. Nevertheless, the overall direction of the findings in this study remains consistent with the wider literature supporting OMP as a useful clinical teaching model.

Student perception findings in the present study were also favorable. Most students viewed OMP positively as a teaching modality, and improvement was observed in several tested items across different domains. This is in line with previous reports showing that OMP promotes active learner participation, encourages self-directed learning, strengthens clinical reasoning, and increases learner satisfaction (Arya et al., 2018; Eckstrom, Homer & Bowen, 2006; Ignoffo et al., 2017; Moin, Sadia & Naqi, 2016; Natesan et al., 2020; Salerno et al., 2002). These findings are also consistent with Cheema, Arora & Kumar (2021), who reported favourable perceptions of OMP and highlighted its usefulness in improving presentation skills, feedback, and assessment-related learning. The favorable student response in the present study is especially relevant for operative dentistry, where the combination of technical performance, clinical reasoning, and immediate supervision makes the quality of chairside teaching particularly important.

### Theoretical implications

The findings of the present study also have theoretical implications for the use of the OMP as a structured microskills model of clinical teaching. OMP was originally conceptualized as a framework to organize brief clinical encounters through learner commitment, probing for supporting evidence, teaching general principles, reinforcing correct actions, and correcting mistakes (Neher et al., 1992; Neher & Stevens, 2003). The present findings are consistent with this framework and suggest that, in operative dentistry clinical settings, OMP may support not only efficient teaching, but also the integration of clinical reasoning, procedural learning, and immediate feedback during chairside encounters. An important contribution of the present study is that it extends the relevance of OMP into a discipline-specific procedural context. Much of the earlier literature has discussed OMP in medicine and other health-professions education as a model for focused teaching and clinical reasoning during short encounters (Aagaard, Teherani & Irby, 2004; Gatewood & De Gagne, 2019; Irby, Aagaard & Teherani, 2004). In the present study, the findings suggest that the model may also be applicable in operative dentistry, where students are required to combine cognitive decision-making with stepwise technical performance in busy clinical environments. This broadens the understanding of OMP from a general clinical teaching tool to a framework that may also be useful in procedural dental education. The findings also provide insight into how different components of the OMP framework may function in this setting. Greater improvement in domains related to learner commitment, probing, and feedback supports the view that OMP is particularly effective in promoting active learner participation and formative feedback during brief clinical encounters (Furney et al., 2001; Moin, Sadia & Naqi, 2016; Shambharkar et al., 2021). At the same time, the relatively limited improvement in some items related to general rules and selected procedural aspects suggests that not all domains may respond equally within a short intervention period. This indicates that some components of professional behavior, counselling, and finer procedural skills may require longer exposure, repeated reinforcement, or additional teaching strategies. In this way, the present study not only supports the OMP model, but also refines its application in procedural dental education.

### Practical implications

The findings of the present study suggest that OMP has practical value for undergraduate operative dentistry teaching in busy clinical settings. Since students in the intervention group showed improvement across several assessed domains and also reported favorable perceptions of OMP, the model appears to provide a feasible framework for structuring brief chairside teaching encounters. It may help preceptors organize focused interactions, assess student reasoning, and provide immediate feedback during patient care. From an academic perspective, OMP may also be considered in faculty development and clinical teaching improvement initiatives to promote more structured, consistent, and learner-centered supervision without requiring major curricular changes.

### Limitations and future research recommendations

The findings of the present study should be interpreted in light of certain limitations. The relatively small sample size and single-center design may limit the generalizability of the findings to other institutions and learner populations. Although simple randomization was used for group allocation, the study was conducted in a single institution in a routine educational setting and did not include blinding or allocation concealment. In addition, the study did not include long-term follow-up, so the durability of the observed gains remains uncertain, and student perceptions were based on self-report, which may be influenced by response bias. Future research should include larger and more diverse samples, multicenter designs, longer follow-up periods, and more objective measures of clinical performance to better evaluate the effectiveness and broader applicability of the OMP model in dental education.

## Conclusion

The findings of this study suggest that the OMP model may be a useful teaching and learning approach for operative dentistry clinical settings, with improvement observed in several assessed student performance domains and positive student perceptions regarding its applicability in busy clinical environments. However, these findings should be interpreted cautiously because of the small sample size, single-center design, and limitations in statistical reporting. Further studies with larger samples and more robust statistical analyses are warranted.

### Recommendations

OMP appears to be highly relevant in the context of increasing workload and busy clinical settings. Therefore, based on the findings of the current study and similar studies in the literature that along with traditional teaching, OMP can be supplemented to improve the analytical and clinical skills of the students. OMP needs to be introduced in all dental schools in Saudi Arabia and similar studies with large sample size should be carried out to assess its utility.

### Lessons for practice

Integrating the OMP model into dental education enhances clinical teaching effectiveness and student competency. Faculty sensitization workshops are crucial for successfully implementing OMP and improving teaching outcomes. OMP promotes active learning and critical thinking among dental students, making it a valuable addition to traditional teaching methods. Regular assessment and feedback on OMP implementation can help refine the approach and maximize its benefits. Dental education should consider adopting OMP as a standard teaching strategy to improve clinical training quality.

##  Supplemental Information

10.7717/peerj.21461/supp-1 Supplemental Information 1 Dataset

## Additional Information and Declarations

### Data Availability

The following information was supplied regarding data availability: The raw data is available in the Supplementary File.
